# Ginsenoside Rg1 Alleviates Podocyte Injury Induced by Hyperlipidemia via Targeting the mTOR/NF-*κ*B/NLRP3 Axis

**DOI:** 10.1155/2020/2735714

**Published:** 2020-10-09

**Authors:** Tao Wang, Yanbin Gao, Rongchuan Yue, Xiaolei Wang, Yimin Shi, Jiayi Xu, Bingjie Wu, Yimeng Li

**Affiliations:** ^1^School of Traditional Chinese Medicine, Capital Medical University, 10 Youanmenwai, Xitoutiao, Fengtai District, Beijing, China; ^2^School of North Sichuan Medical College, Nanchong 637000, China; ^3^Beijing Key Lab of TCM Collateral Disease Theory Research, 10 Youanmenwai, Xitoutiao, Fengtai District, Beijing, China; ^4^Department of Cardiology, Affiliated Hospital of North Sichuan Medical College, Nanchong 637000, China

## Abstract

**Background:**

Podocyte injury plays an important role in diabetic nephropathy (DN). The aim of this study was to determine the potential therapeutic effects of the ginsenoside Rg1 on hyperlipidemia-stressed podocytes and elucidate the underlying mechanisms.

**Methods:**

*In vitro* and *in vivo* models of DN were established as previously described, and the expression levels of relevant markers were analyzed by Western blotting, real-time Polymerase Chain Reaction (PCR), immunofluorescence, and immunohistochemistry.

**Results:**

Ginsenoside Rg1 alleviated pyroptosis in podocytes cultured under hyperlipidemic conditions, as well as in the renal tissues of diabetic rats, and downregulated the mammalian target of rapamycin (mTOR)/NF-*κ*B pathway. In addition, Rg1 also inhibited hyperlipidemia-induced NLRP3 inflammasome in the podocytes, which was abrogated by the mTOR activator L-leucine (LEU). The antipyroptotic effects of Rg1 manifested as improved renal function in the DN rats.

**Conclusion:**

Ginsenoside Rg1 protects podocytes from hyperlipidemia-induced damage by inhibiting pyroptosis through the mTOR/NF-*κ*B/NLRP3 axis, indicating a potential therapeutic function in DN.

## 1. Introduction

Diabetic nephropathy (DN) is a progressive microvascular complication of diabetes mellitus (DM), and the primary cause of end-stage renal diseases (ESRD) [1]. Although the exact mechanism is unclear, several studies have implicated podocyte injury as the key etiological factor [2–4]. Podocytes are specialized glomerular epithelial cells that form the outermost filtration layer, and their loss/damage is a hallmark of various reno-pathological conditions including DN [5, 6]. Pyroptosis, a type of programmed cell death characterized by swelling, pore formation, and release of proinflammatory cytokines (interleukin- (IL-) 1*β* and IL-18), is a potential mechanism of podocyte loss. In fact, pyroptosis has been observed during the development of acute kidney disease and DN, although the specific mechanism is unknown [7].

The pyroptotic cascade is triggered by the nucleotide oligomerization domain- (NOD-) like receptor family pyrin domain containing 3 (NLRP3) inflammasome, a multiprotein complex consisting of NLRP3, apoptosis-associated speck-like protein (ASC) with caspase recruitment domain (CARD) domain and procaspase-1 [8, 9], and the downstream inflammatory pathway is driven by caspase-1 [10, 11]. NLRP3 is activated by mTOR, a highly conserved serine/threonine kinase (Ser/Thr) that regulates cell growth and proliferation [12], in different *in vivo* and *in vitro* models [13, 14]. Recent studies show that high glucose and fat levels can activate NLRP3 inflammasomes and caspase-1 triggered pyroptosis [15, 16]. The inflammatory mTOR-NLRP3-IL-1*β* axis is activated during lung injury, and its inhibition can reduce the immunopathological damage in lung tissues [17]. The nuclear factor kappa-B (NF-*κ*B) and mTOR signaling pathways are highly interrelated [18, 19]. While overexpression of mTOR activates NF-*κ*B, inhibition of mTOR phosphorylation by rapamycin blocks the AKT/NF-*κ*B pathway [20]. In a previous study, we found that inhibiting NF-*κ*B can protect podocytes and reverse DN by blocking mesangial transdermal differentiation [21]. Therefore, we hypothesized that the mTOR-NLRP3-IL-1*β* axis is a potential target for inhibiting podocyte injury in DN.

Ginseng has been used in traditional Chinese medicine formulations for over 2000 years, and has antitransdifferentiation, antioxidation, antiapoptosis, and proautophagy effects [22–24]. The pharmacological effects of ginseng are mainly attributed to the ginsenoside Rg1, which can effectively inhibit apoptosis and protect podocytes [25, 26]. Rg1 also induces autophagy and inhibits apoptosis by activating the AMPK/mTOR pathway [27]. In this study, we analyzed the role of pyroptosis in DN and the therapeutic effects of Rg1.

## 2. Materials and Methods

### 2.1. Main Reagents

**Figure 1:**
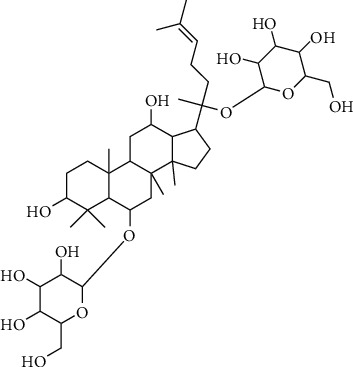
Chemical structure of Ginsenoside Rg1.

Ginsenoside Rg1 (Figure 1, C_42_H_72_O_14_, molecular weight = 801.01, purity by high-performance liquid chromatography (HPLC) ≥ 98%) was purchased from Solarbio (China), and z-YVAD-FMK (C_31_H_39_FN_4_O_9_, MW: 630.66, purity: ≥98%) from ApexBio Technology LLC (USA). Streptozotocin (STZ), 4-methyl-N1-(3-phenylpropyl)-1,2-benzenediamine (JSH-23) and L-leucine (LEU, C_6_H_13_NO_2_, MW: 131.17, purity: ≥98%) were purchased from Sigma-Aldrich (St. Louis, MO, USA). Rapamycin was bought from Selleck Chemicals. Sodium palmitate was purchase from Mecen Unicreate (Beijing, China).

### 2.2. Cell Culture

The conditionally immortalized mouse podocyte cell line BNCC337685 was obtained from BeNa Culture Collection (Beijing, China). The cells were cultured in low glucose DMEM (Genview, Florida, USA) supplemented with 10% fetal bovine serum (FBS; Gibco, Carlsbad, CA, USA) and recombinant IFN-*γ* (PeproTech, London, UK) at 33°C for proliferation, and without IFN-*γ* at 37°C for more than 7 days for inducing differentiation. The cells were harvested at ∼80% confluency and maintained in serum-free conditions for the different assays.

### 2.3. Proliferation Assay

The podocytes were seeded in 96-well plates in serum-free DMEM at the density of 8 × 10^4^ per well and cultured for 24 h. Hyperlipidemia was induced with 100 *μ*M palmitate (PA) in the absence or presence of 0, 10, 25, 50, 75, or 100 *μ*M Rg1 for 48 h [28]. The suitably treated cells were then incubated for an additional 2 h with cell-counting kit-8 (CCK-8, KeyGEN BioTECH, Nanjing, China) solution (10 *µ*L/well), and the optical density of each well was measured at 450 nm. A decrease in cell viability to 30–40% was indicative of podocyte injury [29, 30].

### 2.4. Small Interfering RNA (siRNA) Transfection

The NLRP3-specific (si-NLRP3) and scrambled siRNAs (si-con) were designed and synthesized by RiboBio Co. Ltd. The podocytes were transfected according to the manufacturer's protocol and analyzed for NLRP3 levels later. The NLRP3-targeting siRNA sequence was GTACTTAAATCGTGAAACA.

### 2.5. Establishment of DN Model

SPF-grade male Sprague-Dawley rats (aged 8 weeks, weighing 180–200 g) were purchased from the Beijing Vital River Laboratory Animal Technology Co. Ltd. Diabetes was induced by intraperitoneally injecting the animals once with 50 mg/kg STZ (Sigma-Aldrich, St. Louis, MO, USA) in sodium citrate buffer. The placebo/normal controls (NC, *n* = 8) were injected with an equal volume of 0.1 M citrate buffer, pH 4.5. Serum glucose levels were measured 3 days after injection after drawing blood from the tail veins. Fasting glucose levels ≥16.7 mM for 3 consecutive days were indicative of diabetes [31, 32]. The diabetic rats were fed the high fat diet (HFD; 10% lard, 20% sucrose, 2.5% cholesterol, 0.5% sodium cholate, and 67% basic feed) for 4 weeks to induce DN, while the control rats were given normal food. Four weeks after STZ injection, the successfully modelled DN rats were randomly divided into the (untreated) DN and ginsenoside Rg1-treated groups (*n* = 8 in each group) and administered with vehicle (aqua distillate) or 50 mg/kg ginsenoside Rg1 once daily by oral gavage for 8 weeks. This dose of ginsenoside Rg1 has been proved to be effective and safe [33]. At the end of the regimen, the rats were anaesthetized adequately; then, the urine creatinine (UCr), urinary microalbumin (Malb), blood urea nitrogen (BUN), and serum creatinine (Scr) levels were measured. The rats were then euthanized, and their renal cortices were harvested for further analyses. All animal experiments were approved by the Institutional Animal Care and Use Committee at Capital Medical University and conformed to the Guidelines for the Care and Use of Laboratory Animals by the National Institute of Health.

### 2.6. Histological Analysis

Freshly extracted renal tissues were fixed in 4% paraformaldehyde (KeyGEN BioTECH, Nanjing, China), embedded in paraffin, and cut into 4 *µ*m thick sections. Hematoxylin-eosin (HE), periodic acid-Schiff (PAS), and Masson's staining were performed as per standard protocols, and the tissue sections were observed under a light microscope (Leica DM60008, Kyoto, Japan). For transmission electron microscopy (TEM), the renal cortices were fixed in 2% glutaraldehyde (Servicebio, Wuhan, China), cut into ultrathin sections, and stained accordingly [34]. The samples were observed under a TEM (JEM-1400 plus, JEOL, Tokyo, Japan).

### 2.7. Biochemical Assays

The levels of serum creatinine, urine creatinine, and urine albumin were measured using specific kits from Leadman Biochemistry (Beijing, China) in accordance with the manufacturer's instructions. The biochemical parameters were detected with an automatic chemistry analyzer (7600, HITACHI, Tokyo, Japan). The urinary protein content was calculated relative to the creatinine levels.

### 2.8. Immunofluorescence (IF) and Phalloidin Staining

The paraffin sections were deparaffinized, dehydrated, and heated in citrate buffer for antigen retrieval. After blocking with 10% goat serum (ZSGB-BIO, Beijing, China) for 1 h, the sections were incubated overnight with rabbit antinephrin (1 : 50, sc-377246, Santa Cruz Biotech, CA, USA) antibody and rabbit anti-caspase-1 antibody (1 : 200, ab1872) at 4°C, followed by FITC-conjugated goat anti-rabbit secondary antibody (1 : 100; clone ZF-0311, ZSGB-BIO, Beijing) for 1 h at 37°C. The sections were counterstained with 4′, 6′-diamidino-2-phenylindole (DAPI) (Genview, Florida, USA) and imaged using a laser confocal microscope (TCS SP8 STED, Leica, Wetzlar, Germany). Alternatively, the podocytes were permeabilized with Triton X-100, washed thrice with PBS, and stained with 100 *μ*g/mL phalloidin-conjugate working solution (AAT Bioquest, Sunnyvale, CA, USA) for 1 h at room temperature. The stained cells were washed thrice with PBS and observed under a laser scanning confocal microscope.

### 2.9. Immunohistochemical Staining

The paraffin-embedded sections were processed via routine dewaxing and hydration. In brief, sections were heated in a microwave for antigen retrieval and blocked with 3% H_2_O_2_ (ZSGB-BIO, Beijing, China) for 10 min to avoid nonspecific binding. The sections were subsequently incubated with anti-ASC antibody (1 : 100, ab47092, abcam, UK) at 4°C overnight and reacted with poly peroxidase-anti-mouse IgG (ZSGB-BIO, Beijng, China). Afterwards, 3,3-diaminobenzidine (DAB, ZSGB-BIO) was used for visualization of immunoreactive signals. Finally, the sections were observed under a microscope (Leica DM60008) at 400x magnifications.

### 2.10. TUNEL Assay

Tissue sections and podocytes cultured on coverslips were treated with proteinase K (20 g/mL) and stained using the In-Situ Cell Death Detection kit (Nanjing KeyGen Biotech Co. Ltd., China) according to the manufacturer's instructions. The number of TUNEL-positive cells was counted in 10 randomly selected, nonoverlapping fields at 200x magnification. Apoptotic index was calculated as the number of TUNEL-positive cells per 10^3^ cells and %TUNEL-positive podocytes.

### 2.11. Western Blotting

The total protein of cultured podocytes and kidney tissues were extracted, separated by SDS-PAGE, and then transferred to PVDF membranes. After blocking with 5% skim milk in PBS + 0.05% tween 20, the blots were incubated overnight with rabbit polyclonal antibodies against procaspase-1 (1 : 1000, ab179515), IL-1*β* (1 : 500, ab9722), mTOR (1 : 1000, ab2732), NLRP3 (1 : 500, ab214185), ASC (1 : 500, ab47092), caspase-1 p20 (Casp1 p20, 1 : 500, ab1872) (all from Abcam, Cambridge, UK) and p-mTOR (1 : 500, 5536), rabbit monoclonal antibodies targeting NF-*κ*B p65 (p-65, 1 : 1000, 8242) and p-NF-*κ*B p65 (p-p65, 1 : 500, 3033) (all from Cell Signaling Technology, Inc., Danvers, MA, USA), and mouse monoclonal antinephrin (1 : 500, sc-377246) and anti-GAPDH (1 : 2000, 60004-1-lg; Proteintech Group, Rosemont, USA) antibodies at 4°C. The blots were then incubated with HRP goat anti-rabbit IgG (RS0002) or HRP goat anti-mouse IgG (RS0001) secondary antibodies (both diluted 1 : 5000, ImmunoWay Biotechnology, TX, USA) as appropriate. The positive bands were developed using a chemiluminescent reagent (Thermo Scientific, USA).

### 2.12. Reverse Transcription Quantitative Polymerase Chain Reaction (RT-qPCR)

Total RNA was isolated from the cultured podocytes and renal cortices using TRIzol Reagent according to the manufacturer's instructions and reverse-transcribed using the PrimeScript™ RT kit. The qRT-PCR analysis was performed using the SYBR® Premix Ex Taq™ II (Takara) in the ABI PRISM 7500 FAST Real-TIME PCR System (ABI, Vernon, CA, USA). Relative gene expression levels were calculated by the 2ΔCT method. The sequence of mTOR primers was as follows: forward: 5′-AGTGAAGCCGAGAGCAATGAGA-3′ and reverse: 5′-GACAAGGAGATAGAACGGAAGAAGC-3′.

### 2.13. Propidium Iodide (PI)/Hoechst 33342 Double Staining

The suitably treated podocytes were harvested and stained with Hoechst 33342 at 37°C for 10 minutes. The cells were washed with PBS 3 times, and then the 1 × Buffer and the PI were added to stain the podocytes in the dark for 10 minutes. The cells were observed under an inverted fluorescence microscope (Carl Zeiss, Oberkochen, Germany). Whether the PI could penetrate the pyroptosis cell membrane and enter into the nucleus was determined by the appearance of red fluorescence, indicating the presence of dead cells as a result of pyroptosis. The pyroptosis rate was calculated as the number of dead cells/the total number of cells × 100%. The experiment was conducted in triplicate.

### 2.14. Statistical Analysis

The data were presented as mean ± standard deviation (SD). Statistical analysis was performed using the SPSS 19.0 software (IBM Corporation, Armonk, NY, USA). Student's *t*-test was used to compare the difference between two groups, while multiple groups were compared by one-way analysis of variance (ANOVA). A *P* value <0.05 was considered statistically significant.

## 3. Results and Discussion

### 3.1. Ginsenoside Rg1 Protects Podocytes against Hyperlipidemia-Induced Injury

**Figure 2:**
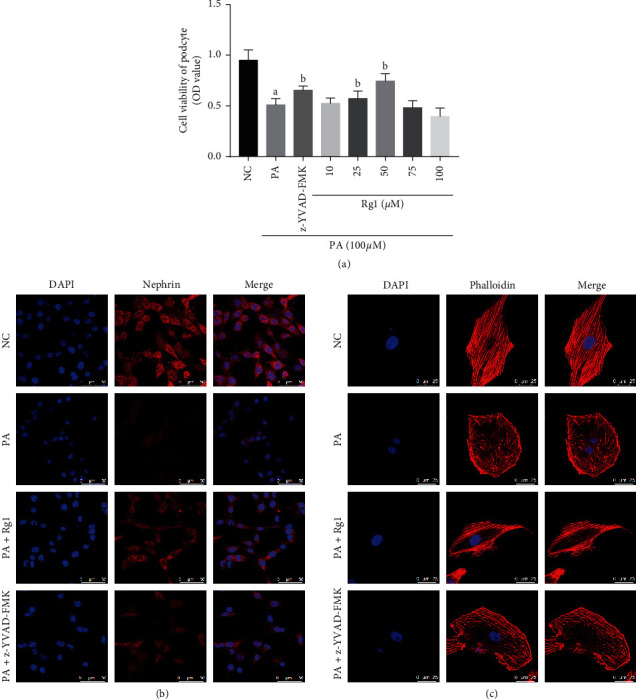
Protective effect of Rg1 on hyperlipidemia-treated podocytes. (a) The proliferation rate of podocytes exposed to hyperlipidemic conditions with/without Rg1 or z-YVAD-FMK for 48 h. (b) Representative images of immunofluorescent staining showing nephrin expression in cultured podocytes (original magnification 630x). (c) Representative images of podocytes with phalloidin-stained cytoskeleton (original magnification 630x). Values are the mean ± SD; *n* = 3. ^a^*P* < 0.05 compared to the NC group, ^b^*P* < 0.05 compared to the PA group. Abbreviations: PA, palmitate group; Rg1, ginsenoside Rg1 group; NC, normal control; DN, diabetic nephropathy group; DAPI, 4′,6-diamidino-2-phenylindole.

**Figure 3:**
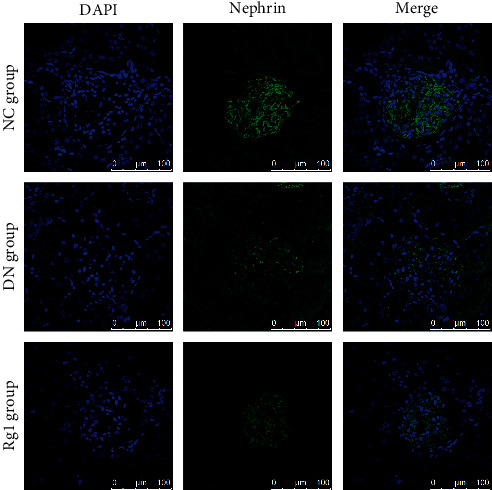
Effect of Rg1 on nephrin expression of DN rats: representative images of immunofluorescent staining showing *in situ* nephrin expression in renal tissues. Blue, nuclear staining (DAPI); green, target protein staining (original magnification 630x, *n* = 3).

The optimal therapeutic dose of Rg1 was determined in an *in vitro* model of podocyte injury. As shown in Figure 2(a), Rg1 increased the viability of podocytes in a dose-dependent manner at lower concentrations for 48 h, while doses above 50 *μ*M were cytotoxic. Therefore, 50 *μ*M Rg1 for 48 h was used for subsequent experiments. Podocytes exposed to hyperlipidemic conditions showed reduced activity and low expression level of nephrin, while these effects were restored by Rg1 treatment (Figures 2(a) and 2(b)). Consistent with the above observation *in vitro*, in normal group rats, nephrin staining presented a green linear-like pattern along the capillary loops of glomeruli. Instead, a light green, discontinuous, short linear-like or punctiform staining pattern of nephrin was observed in DN group, and Rg1 also upregulated the *in situ* expression of nephrin in the glomeruli of DN rats (Figure 3).

*In vitro*, actin stress fibers were arranged regularly or parallel to each other in podocytes of normal group. Phalloidin staining further revealed the collapsed or totally absent intracellular actin stress fibers in the hyperlipidemia-treated podocytes, resulting in the polygonal cells. Ginsenoside Rg1 treatment remodeled the podocyte skeleton and restored their normal shape (Figure 2(c)).

Taken together, Rg1 protects podocytes against hyperlipidemia-induced injury by restoring nephrin levels and the cytoskeleton.

### 3.2. Ginsenoside Rg1 Alleviated Hyperlipidemia-Induced Pyroptosis in Podocytes

**Figure 4:**
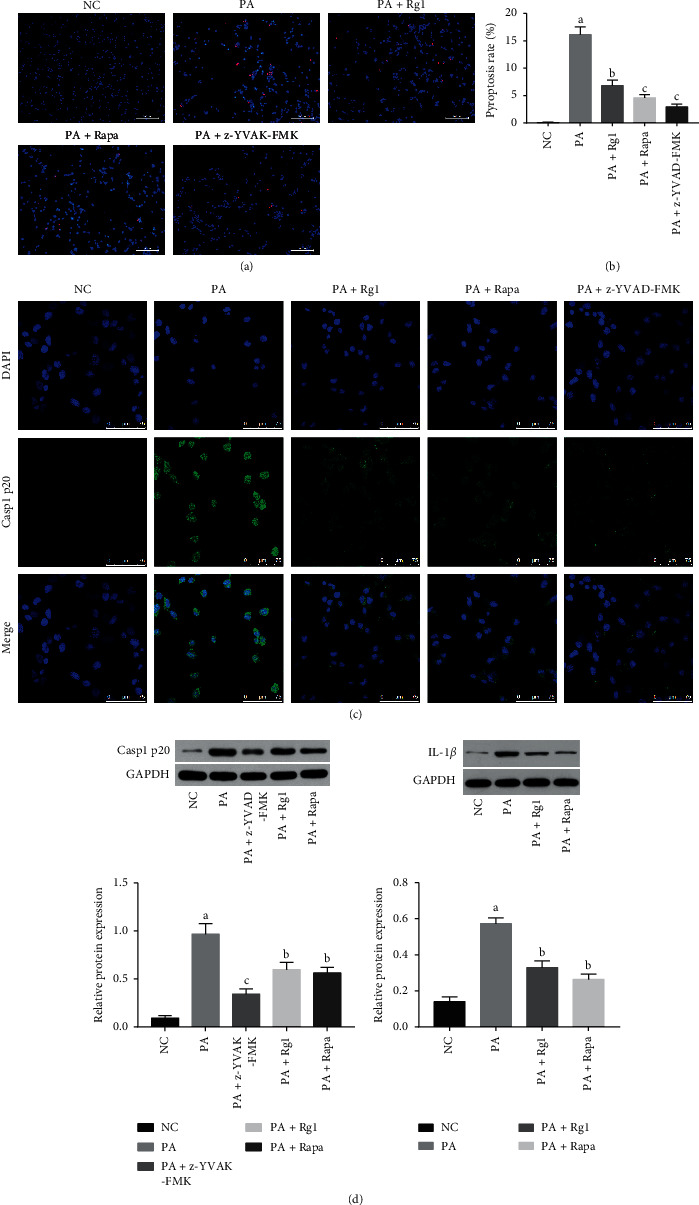
Effect of Rg1 on hyperlipidemia-induced pyroptosis in podocytes. (a)-(b) Representative PI/Hoechst 33342-stained images showing pyroptotic podocytes. Red fluorescence was regarded as PI-positive podocytes, indicating the presence of dead cells as a result of pyroptosis (original magnification 200x). (c) Representative immunofluorescent images showing *in situ* Casp1 p20 expression in the suitably treated podocytes (original magnification 630x). (d) Immunoblot showing total Casp1 p20 and IL-1*β* levels in the suitably treated podocytes. Values are the mean ± SD; *n* = 3. ^a^*P* < 0.05 compared to the NC group, ^b^*P* < 0.05 compared to the PA group, ^c^*P* < 0.05 compared to the PA + Rg1 group. Abbreviations: Rapa, rapamycin.

The positive rate of PI staining revealed that, compared with the NC group, there was significant increased pyroptosis rate in PA-induced podocytes (Figures 4(a) and 4(b)). Pyroptosis is also characterized by caspase-1 activation and IL-1*β* secretion [35]. As shown in Figures 4(c) and 4(d), the expression of Casp1 p20 and IL-1*β* was markedly increased in the hyperlipidemia-stressed podocytes. Consistent with this, pretreatment of the hyperlipidemia-stressed podocytes with 20 *μ*M caspase-1 inhibitor z-YVAD-FMK significantly increased cell viability and nephrin levels, decreased the rate of PI-positive podocytes, and restored the cytoskeletal structure (Figures 2(a)–2(c), 4(a) and 4(b)), thus further confirming that hyperlipidemia-induced podocyte injury *in vitro* was partly mediated via pyroptosis induction. Furthermore, Rg1 significantly decreased pyroptosis rates in the suitably treated podocytes, which was also correlated to markedly lower levels of Casp1 p20 and IL-1*β* compared to the PA group (Figures 4(a)–4(d)). However, the pyroptosis rate and the level of Casp1 p20 of Rg1 group were higher than those in the PA + z-YVAD-FMK groups; these results also strongly suggested that hyperlipidemia induced pyroptosis of podocytes, which was alleviated by Rg1.

**Figure 5:**
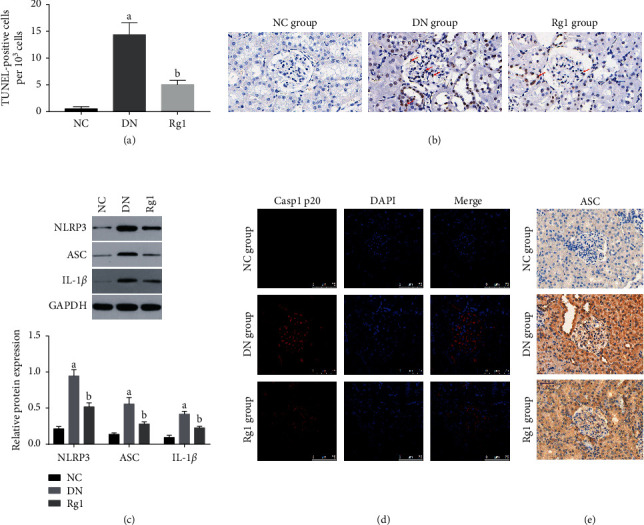
Rg1 inhibited NLRP3 and alleviated cell death in diabetic renal tissues. (a)-(b) Representative TUNEL images showing apoptotic cells in the renal tissue (original magnification 400x); red arrows point to the TUNEL-positive cells. (c) Immunoblot showing NLRP3, ASC, and IL-1*β* levels in the renal tissues of the different groups. (d) Representative immunofluorescent images showing *in situ* Casp1 p20 expression in renal tissues. Blue, nuclear staining (DAPI); red, target protein staining (original magnification 630x). (e) Representative immunohistochemical staining showing *in situ* ASC expression in renal tissues. Brownish or yellowish deposits were regarded as positive immunostaining (original magnification 400x). Values are the mean ± SD; *n* = 3. ^a^*P* < 0.05 compared with the NC group, ^b^*P* < 0.05 compared with the PA group. Abbreviations: NLRP3, nucleotide-binding oligomerization domain-like receptor with a pyrin domain (NLRP) 3; ASC, apoptosis-associated speck-like protein.

Moreover, increased TUNEL-positive cells were observed in the glomeruli and renal tubules of the DN rats, which was significantly alleviated by Rg1 treatment (Figures 5(a) and 5(b)). Immunofluorescence images and immunoblot showed that the glomeruli of the untreated DN rats also expressed higher levels of activated caspase-1 (Casp1 p20) and IL-1*β*, which was decreased by Rg1 (Figures 5(c) and 5(d)).

### 3.3. Ginsenoside Rg1 Targets the mTOR/NF-*κ*B Pathway in Podocytes under Hyperlipidemic Conditions

**Figure 6:**
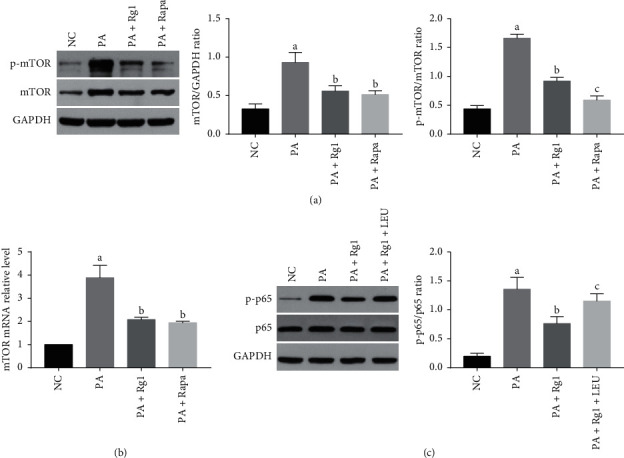
Rg1 inhibits mTOR and NF-*κ*B p65 expression in the podocytes *in vitro*. (a) Immunoblot showing mTOR and p-mTOR levels in the suitably treated podocytes. (b) The mTOR mRNA levels in the podocytes treated as indicated. (c) Immunoblot showing p-p65 and p65 levels in the suitably treated podocytes. Values are the mean ± SD; *n* = 3. ^a^*P* < 0.05 compared with the NC group, ^b^*P* < 0.05 compared with the PA group, ^c^*P* < 0.05 compared with the PA + Rg1 group.

**Figure 7:**
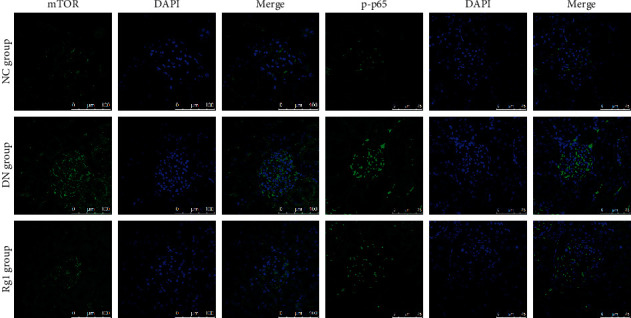
Rg1 inhibits mTOR and NF-*κ*B p65 expression in diabetic renal tissues. Representative immunofluorescence images showing *in situ* mTOR and p-p65 expression in the renal tissues. Blue, nuclear staining (DAPI); green, target protein staining (original magnification 630x, *n* = 3).

Studies have implicated mTOR in podocytes injury and DN [36, 37]. Consistent with this, hyperlipidemia treatment significantly increased total and phosphorylated mTOR levels in the cultured podocytes (Figures 6(a) and 6(b)), as well as in renal tissues of diabetic rats (Figure 7). Furthermore, pretreatment with the mTOR inhibitor rapamycin (10 ng/mL) decreased total and phosphorylated mTOR levels (Figures 6(a) and 6(b)), inhibited the expression of Casp1 p20 and IL-1*β*, and also abrogated hyperlipidemia-induced pyroptosis (Figure 4). Rg1 significantly downregulated mTOR and p-mTOR (Figure 6(a)), as well as p-p65 (Figure 6(c)) in the hyperlipidemic podocytes. However, pretreatment of these podocytes with the mTOR activator LEU (5 mM) abrogated the suppressive effects of Rg1 on p-p65 (Figure 6(c)). Therefore, the mTOR/NF-*κ*B pathway may be one of the effect targets of Rg1 on the PA-induced podocytes.

Similarly, mTOR and p-p65 were significantly upregulated in the renal tissues of untreated DN rats compared to the NC group and decreased following Rg1 treatment (Figure 7).

### 3.4. Rg1 Inhibited Hyperlipidemia-Induced NLRP3 in Podocytes by Suppressing the mTOR/NF-*κ*B Pathway

**Figure 8:**
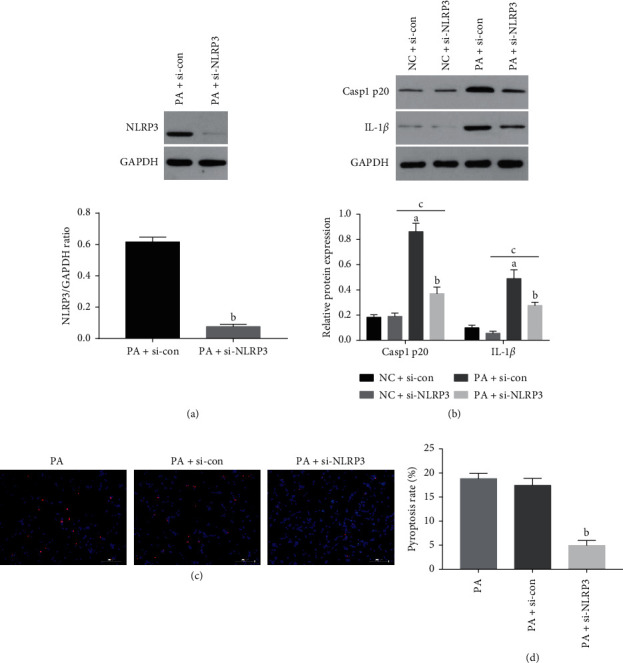
Downregulation of NLRP3 inhibited pyroptosis in podocytes under hyperlipidemic conditions. The podocytes were treated with si-con or si-NLRP3 in the presence (or absence) of PA for 48 h. (a) NLRP3 and (b) Casp1 p20 and IL-1*β* levels in the suitably treated cells. (c)-(d) Representative PI/Hoechst 33342-stained images showing pyroptotic podocytes (original magnification 200x). Values are the mean ± SD; *n* = 3. ^a^*P* < 0.05 compared with the NC + si-con group, ^b^*P* < 0.05 compared with the PA + si-con group, ^c^*P* < 0.05 compared with the NC + si-NLRP3 group. Abbreviations: si-NLRP3, the podocytes transfected by the NLRP3-specific siRNA; si-con, the podocytes transfected by scrambled siRNA.

Since several inflammasomes can trigger pyroptosis, we silenced NLRP3 in the podocytes in order to determine whether NLRP3 inflammasome is essential for hyperlipidemia-induced pyroptosis in podocytes. As shown in Figure 8, NLRP3 knockdown (Figure 8(a)) significantly reduced the pyroptosis rate (Figure 8(c)) and the expression of Casp1 p20 and IL-1*β* in the podocytes exposed to hyperlipidemic conditions (Figure 8(b)). This suggests that podocyte pyroptosis induced by hyperlipidemia was mainly regulated by NLRP3 inflammasome.

**Figure 9:**
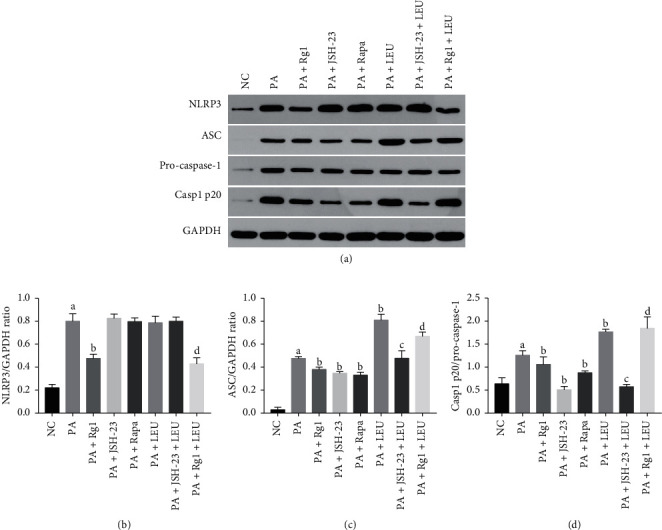
Rg1 inhibited the pyroptotic cascade in podocytes via the mTOR/NF-*κ*B/NLRP3 axis: immunoblot and quantification of NLRP3, ASC, procaspase-1, and Casp1 p20 in podocytes treated as indicated. Values are the mean ± SD; *n* = 3. ^a^*P* < 0.05 compared with the NC group, ^b^*P* < 0.05 compared with the PA group, ^c^*P* < 0.05 compared with the PA + LEU group, ^d^*P* < 0.05 compared with the PA + Rg1 group.

Previous studies have shown that NLRP3 is activated by mTOR/NF-*κ*B [13, 38]. Consistent with this, the NF-*κ*B inhibitor JSH-23 (20 *μ*M) and rapamycin significantly decreased the expression of ASC and Casp1 p20 in the hyperlipidemic podocytes (Figure 9), which were significantly elevated by LEU. Interestingly, JSH-23 neutralized the activation effects of LEU on NLRP3 under hyperlipidemic conditions (Figure 9). Furthermore, Rg1 markedly inhibited the expression and activation of NLRP3 under hyperlipidemic conditions; however, the inhibitory effect of Rg1 on NLRP3 was attenuated by LEU (Figure 9). NLRP3, ASC, and Casp1 p20 levels were also upregulated in the glomeruli of DN rats and deceased upon Rg1 treatment (Figures 5(c)–5(e)). These findings indicated that hyperlipidemia-induced NLRP3 activation was dependent on the mTOR/NF-*κ*B pathway, and Rg1 targeted the mTOR/NF-*κ*B/NLRP3 axis to alleviate pyroptosis in the podocytes.

### 3.5. Effect of Ginsenoside Rg1 on Renal Function and Morphology in Diabetic Rats

**Figure 10:**
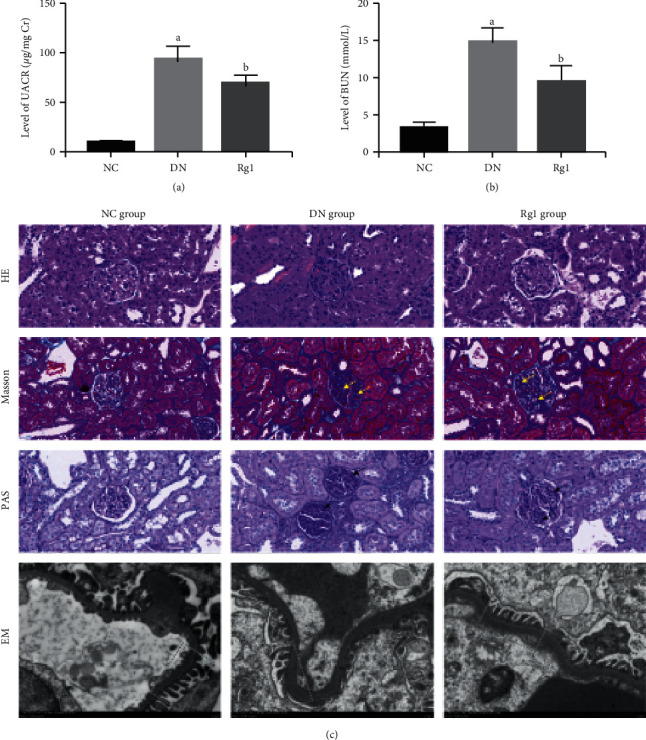
Rg1 improved renal function in DN rats. (a) and (b) UACR and BUN levels in the untreated and Rg1-treated DN rats. (c) Representative HE, PAS, Masson (original magnification 200x), and EM (original magnification 10,000x) images showing structure of the glomerular basement membrane (GBM) in the different groups. Values are the mean ± SD; *n* = 3. ^a^*P* < 0.05 compared with the NC group, ^b^*P* < 0.05 compared with the PA group. The yellow arrows show mesangial matrix. The black arrows show glycogen storage. The dotted yellow boxes indicate the GBM. Abbreviations: UACR, urine albumin creatinine ratio; BUN, blood urea nitrogen; HE, hematoxylin-eosin; PAS, periodic acid-Schiff; EM, electron microscope.

The therapeutic effects of ginsenoside Rg1 on renal function and morphology were measured in terms of standard biochemical and histopathological indices. Rg1 significantly decreased UACR and BUN levels compared to those in untreated DN rats (Figures 10(a) and 10(b)). In addition, kidney tissues were collected for routine HE, PAS, and Masson staining at the end of the experiment. Furthermore, electron microscopy (EM) was used to observe the thickness of glomerular basement membrane (GBM). The results of HE, PAS, and Masson's staining demonstrated that the DN model rats presented obvious pathological changes compared with those of normal group, while Rg1 alleviated glomerular hypertrophy with diffuse and nodular sclerosis, excessive glycogen storage, and ultrastructural anomalies like glomerular podia cell fusion, rupture, and loss in the diabetic rats (Figure 10(c)). Taken together, Rg1 improved renal function and morphology in the DN rats.

## 4. Discussion

The ginsenoside Rg1 is one of the pharmacologically active components of ginseng and exhibits antiapoptotic, anti-inflammatory, and neuroprotective effects [39–42]. It also has a significant reno-protective action [43] and alleviates aldosterone-induced podocyte injury [44]. In line with these findings, we found that Rg1 also protected podocytes from hyperlipidemic injury both *in vivo* and *in vitro*.

Pyroptosis is a form of programmed cell death mediated by the NLRP3 inflammasome and caspase-1 and is the pathological basis of various inflammatory and degenerative disorders [17, 35]. It is frequently induced by hyperlipidemia and therefore associated with diabetic cardiomyopathy and diabetic retinopathy [45, 46]. Recently, Wang et al. detected increased pyroptosis in the tubular cells involved in diabetic kidney disease (DKD) [7]. Similarly, we also observed pyroptosis in podocytes exposed to hyperlipidemic conditions, indicating its involvement in DN. The mTOR pathway has been previously implicated in podocyte injury [47] and was also upregulated in the affected cells in our study. In addition, there is a positive crosstalk between mTOR and NF-*κ*B [48, 49], and Wei et al. associated NF-*κ*B pathway activation with damaged podocytes [50]. Rg1 significantly inhibited pyroptosis, restored nephrin expression and cytoskeletal structure in the podocytes, and blocked hyperlipidemia-induced mTOR/NF-*κ*B signaling.

The pyroptotic cascade is triggered by the NLRP3 inflammasome, which then activates caspase-1 and releases IL-1*β* [51–53]. While IL-1*β* mediates podocyte injury and renal inflammation [54], NLRP3 is involved in the pathogenesis of chronic kidney disease [55, 56]. Since hyperlipidemia can activate multiple inflammasomes [57], we knocked down NLRP3 in podocytes and found that absence of NLRP3 inhibited pyroptosis, indicating that it initiates pyroptosis in podocytes during DN.

Since NF-*κ*B activation is needed to activate the NLRP3 inflammasome [58–60], we also treated the podocytes with mTOR activator/inhibitor and NF-*κ*B inhibitor. JSH23 and rapamycin both attenuated NLRP3 activation, while cells treated with LEU showed high levels of NLRP3, ASC, and Casp1 p20. In addition, JSH-23 abrogated the effect of LEU on the activation of NLRP3, indicating that mTOR activates NLRP3 via NF-*κ*B. Rg1 also significantly decreased NLRP3 levels in the hyperlipidemia-exposed podocytes. Furthermore, the inhibitory effects of Rg1 on the activation of NLRP3 were reversed by LEU, which strongly suggested that Rg1 decreased hyperlipidemia-induced pyroptosis in podocytes by inhibiting the mTOR/NF-*κ*B/NLRP3 axis. A previous study showed that the combination of Rg1 and astragaloside relieved renal fibrosis in DN by inhibiting the TGF-*β*1/Smads pathway and oxidative stress [33]. Consistent with this, Rg1 significantly reduced proteinuria, improved renal function, and alleviated tissue damage in diabetic rats. Podocyte is the functional unit of the glomerular filtration membrane [61], and its injury is the pathological basis of DN [62]. Thus, Rg1-mediated podocyte rescue translated to improved renal function.

In summary, Rg1 improves renal function in DN rats by inhibiting hyperlipidemia-induced pyroptosis in podocytes via targeting mTOR/NF-*κ*B/NLRP3 signaling. Our findings provide the experimental basis for future clinical trials on Rg1 as a treatment option for DN.

## 5. Conclusions

Ginsenoside Rg1 protects podocytes from hyperlipidemia-induced damage by inhibiting pyroptosis through the mTOR/NF-*κ*B/NLRP3 axis, indicating a potential therapeutic function in DN.

## Data Availability

The data used to support the findings of this study are included within the article.
